# New Transposon Tools Tailored for Metabolic Engineering of Gram-Negative Microbial Cell Factories

**DOI:** 10.3389/fbioe.2014.00046

**Published:** 2014-10-28

**Authors:** Esteban Martínez-García, Tomás Aparicio, Víctor de Lorenzo, Pablo I. Nikel

**Affiliations:** ^1^Systems and Synthetic Biology Program, Centro Nacional de Biotecnología (CNB-CSIC), Madrid, Spain

**Keywords:** metabolic engineering, *Pseudomonas putida*, *Escherichia coli*, transposon mini-Tn*5*, central metabolism, chromosomal integration, polyhydroxyalkanoates

## Abstract

Re-programming microorganisms to modify their existing functions and/or to bestow bacteria with entirely new-to-Nature tasks have largely relied so far on specialized molecular biology tools. Such endeavors are not only relevant in the burgeoning metabolic engineering arena but also instrumental to explore the functioning of complex regulatory networks from a fundamental point of view. *À la carte* modification of bacterial genomes thus calls for novel tools to make genetic manipulations easier. We propose the use of a series of new broad-host-range mini-Tn*5*-vectors, termed pBAMDs, for the delivery of gene(s) into the chromosome of Gram-negative bacteria and for generating saturated mutagenesis libraries in gene function studies. These delivery vectors endow the user with the possibility of easy cloning and subsequent insertion of functional cargoes with three different antibiotic-resistance markers (kanamycin, streptomycin, and gentamicin). After validating the pBAMD vectors in the environmental bacterium *Pseudomonas putida* KT2440, their use was also illustrated by inserting the entire poly(3-hydroxybutyrate) (PHB) synthesis pathway from *Cupriavidus necator* in the chromosome of a phosphotransacetylase mutant of *Escherichia coli*. PHB is a completely biodegradable polyester with a number of industrial applications that make it attractive as a potential replacement of oil-based plastics. The non-selective nature of chromosomal insertions of the biosynthetic genes was evidenced by a large landscape of PHB synthesis levels in independent clones. One clone was selected and further characterized as a microbial cell factory for PHB accumulation, and it achieved polymer accumulation levels comparable to those of a plasmid-bearing recombinant. Taken together, our results demonstrate that the new mini-Tn*5*-vectors can be used to confer interesting phenotypes in Gram-negative bacteria that would be very difficult to engineer through direct manipulation of the structural genes.

## Introduction

Over the last few years, a number of Gram-negative bacteria have become increasingly attractive *chassis* for a number of synthetic biology and metabolic engineering purposes. One conspicuous case involves the environmental bacterium *Pseudomonas putida* as a robust host for strong oxidative bioreactions, together with its GRAS (generally recognized as safe) status and its inherent ability to grow on a wide range of substrates (Nikel et al., 2014a). This situation calls for the expansion of the available tools for re-wiring its extant genetic features to further extend its metabolic potential – or even introducing new-to-Nature functions.

One frequently used molecular biology resource for analyses and manipulations of bacterial genomes is the Tn*5* transposon. Historically, a number of plasmid vectors based on both wild-type and minimized versions of Tn*5* (i.e. mini-transposons) had allowed the user to introduce stable insertions of foreign DNA into the chromosome of virtually any Gram-negative bacteria (de Lorenzo et al., 1990, 1998; Herrero et al., 1990; de Lorenzo and Timmis, 1994; Martínez-García et al., 2011; Martínez-García and de Lorenzo, 2012; Nikel and de Lorenzo, 2013a). Such Tn*5*-derived elements present clear advantages over the use of their plasmid-based counterparts for the introduction and expression of heterologous genes into several bacterial species. These features include (but are not limited to) (i) the maintenance of the corresponding transgenes without antibiotic selective pressure, (ii) the long-term stability of the constructs and the re-usability of the functional parts, and, furthermore, (iii) Tn*5* vectors admit cloning and chrosomosomal delivery of considerably long DNA fragments. Finally, as the transposase gene (*tnpA*) is lost following each transposition event (Berg, 1989; Reznikoff, 2006, 2008), one added value of mini-Tn*5*-vectors is the possibility to use them recursively in the same host, provided that they bear different selection markers. Moreover, as the TnpA transposase tends to act in *cis* (Phadnis et al., 1986), it promotes the insertion of DNA sequences borne by the plasmid, irrespective of previous insertions in a given target chromosome. These features allow for the integration of more than one DNA cargo into the same genome.

In this study, we report a series of synthetic, modular broad-host-range mini-Tn*5*-vectors for the delivery of gene(s) into the chromosome of a diversity of Gram-negative bacteria and to construct saturated mutagenesis libraries for gene function studies. These vectors were termed pBAMDs, and they enable the possibility of easy cloning and subsequent chromosomal insertion of functional cargoes with three different and interchangeable antibiotic-resistance markers (kanamycin, streptomycin, and gentamicin). Moreover, the functional parts of the new vectors can be easily swapped by digestion with the appropriate restriction enzymes, allowing for the shuffling of each element as needed. Potential applications of the new tools are illustrated in two different genetic contexts. In one case, a systematic validation of the Tn*5* vectors was carried out in *P*. *putida* KT2440, demonstrating the potential of the new insertional plasmids to be used in a sequential fashion for constructing (and deconstructing) complex phenotypes. In a second case, one of the pBAMD plasmids was used to insert a gene cluster from *Cupriavidus necator*, encoding all the biochemical functions needed for the formation of poly(3-hydroxybutyrate) (PHB), into the chromosome of *Escherichia coli*, thereby resulting in a new microbial cell factory tailored for biopolymer synthesis.

## Materials and Methods

### Bacterial strains, plasmids, and growth conditions

The bacterial strains and plasmids used in this study are described in Table 1. Bacteria were routinely grown batchwise in LB medium (10 g l^−1^ tryptone, 5 g l^−1^ yeast extract, and 5 g l^−1^ NaCl) with rotary agitation (170 rpm). *P*. *putida* was grown at 30°C while *E*. *coli* cells were grown at 37°C. Selection of *P*. *putida* transconjugants was performed by spotting the cells onto M9 minimal medium agar plates (Sambrook et al., 1989) added with 0.2% (w/v) sodium citrate as the sole carbon source (to counterselect *E*. *coli* cells). PHB accumulation was assessed in selected *E*. *coli* transconjugants cultured in M9 minimal medium containing 30 g l^−1^ glucose as the sole carbon source. Aerobic culture conditions in experiments aimed at polymer synthesis were achieved essentially as described by Nikel et al. (2010a), by using a 1:10 culture medium-to-flask volume ratio. Antibiotics were added at the following final concentrations whenever needed: ampicillin, 150 μg ml^−1^ for *E*. *coli* or 500 μg ml^−1^ for *P*. *putida*, chloramphenicol, 30 μg ml^−1^; kanamycin, 50 μg ml^−1^, streptomycin, 80 μg ml^−1^; and gentamicin, 10 μg ml^−1^. All solid media also contained 15 g l^−1^ agar. Growth was estimated spectrophotometrically by measuring the optical density at 600 nm (OD_600_) of the cultures (appropriately diluted in 9 g l^−1^ NaCl whenever needed) in a Ultrospec 3000 *pro* UV/Visible spectrophotometer (GE Healthcare Bio-Sciences Corp., Piscataway, NJ, USA). When culturing *E*. *coli* strains that accumulate PHB, for which OD_600_ readings are no longer useful to estimate the cell dry weight (CDW), cells from 15 ml aliquots were washed, concentrated, and the CDW determined after drying the samples at 80°C to constant weight as previously indicated by Nikel et al. (2008a,b).

**Table 1:** **Bacterial strains and plasmids used in this work**.

Bacterial strain or plasmid	Relevant characteristics^a^	Reference or source
***Escherichia coli***		
DH5α λ*pir*	Cloning host; F^−^λ^−^*endA1 glnX44*(AS) *thiE1 recA1 relA1 spoT1 gyrA96*(Nal^R^) *rfbC1 deoR nupG* Φ80(*lacZ*Δ*M15*) Δ(*argF*-*lac*)*U169 hsdR17*(*r_K_*^−^*m_K_*^+^), λ*pir* lysogen	Hanahan and Meselson (1983)
CC118 λ*pir*	Cloning host; Δ(*ara*-*leu*) *araD* Δ*lacX174 galE galK phoA thiE1 rpsE rpoB*(Rif^R^) *argE*(Am) *recA1*, λ*pir* lysogen	Herrero et al. (1990)
S17-1 λ*pir*	Cloning host; F^−^*recA1 endA1 thiE1 pro-82 creC510 hsdR17* RP4-2(Km:Tn*7* Tc:Mu-1), λ*pir* lysogen, Sm^R^	de Lorenzo et al. (1993)
S17P	Same as S17-1 λ*pir* but carrying plasmid pBAMD1-6-*pha* (*phaC1AB1*^+^), Sm^R^ Ap^R^ Gm^R^	This work
HB101	Helper strain; F^−^λ^−^*hsdS20*(*r_B_*^−^*m_B_*^−^) *recA13 leuB6*(Am) *araC14* Δ(*gpt-proA*)*62 lacY1 galK2*(Oc) *xyl-5 mtl-1 thiE1 rpsL20*(Sm^R^) *glnX44*(AS)	Boyer and Roulland-Dussoix (1969)
BW25113	Wild-type strain; F^−^λ^−^Δ(*araD-araB*)*567* Δ*lacZ4787*(:*rrnB-3*) *rph-1* Δ(*rhaD-rhaB*)*568 hsdR514*	Datsenko and Wanner (2000)
JW2293-1^b^	Same as BW25113 but Δ*pta779*:*aphA*, Km^R^	Baba et al. (2006)
JW2293P	Same as JW2293-1 but carrying plasmid pAeT41 (*phaC1AB1*^+^), Ap^R^	This work
TA2293P	Same as JW2293-1 but *ykgH*:mini-Tn*5*(*phaC1AB1*), expresses the *pha* gene cluster from *Cupriavidus necator* as a chromosomal integration, Km^R^ Gm^R^	This work
***Pseudomonas putida***		
KT2440	Wild-type strain; mt-2 derivative cured of the TOL plasmid pWW0	Bagdasarian et al. (1981)
**Plasmids**		
pRK600	Helper plasmid used for conjugation; *ori*(ColE1), RK2(*mob*^+^ *tra*^+^); Cm^R^	Kessler et al. (1992)
pBAM1	Mini-Tn*5* delivery plasmid; *ori*(R6K), standard multiple cloning site; Ap^R^ Km^R^	Martínez-García et al. (2011)
pSEVA111	Cloning vector; *ori*(R6K), standard multiple cloning site; Ap^R^	Silva-Rocha et al. (2013)
p-R-SETA111	Cloning vector; *ori*(R6K), standard multiple cloning site, *tnpA*; Ap^R^	This work
pBAMD1-2	Mini-Tn*5* delivery plasmid; *ori*(R6K); Ap^R^ Km^R^	This work
pBAMD1-4	Mini-Tn*5* delivery plasmid; *ori*(R6K); Ap^R^ Sm^R^/Sp^R^	This work
pBAMD1-6	Mini-Tn*5* delivery plasmid; *ori*(R6K); Ap^R^ Gm^R^	This work
pAeT41	Derivative of cloning vector pUC18 (Thermo Fisher Scientific Inc.; Pittsburgh, PA, USA) bearing a *ca*. 5-kb *Sma*I/*Eco*RI DNA fragment spanning the *phaC1AB1* gene cluster from *C*. *necator*; Ap^R^	Peoples and Sinskey (1989)
pBAMD1-6-*pha*	Derivative of pBAMD1-6; delivery plasmid used to insert the *phaC1AB1* gene cluster from *C*. *necator* into the chromosome of recipient *E*. *coli* strains; Ap^R^ Gm^R^	This work

*^a^Antibiotic markers: Ap, ampicillin; Cm, chloramphenicol; Gm, gentamicin; Km, kanamycin; Nal, nalidixic acid; Rif, rifampicin; Sm, streptomycin; Sp, spectinomycin; and Tc, tetracycline*.

*^b^Strain obtained from the *E*. *coli* Genetic Stock Center (Yale University, New Haven, CT, USA)*.

### Nucleic acid manipulations and general cloning techniques

DNA manipulations followed routine laboratory techniques as described by Sambrook et al. (1989) and Martínez-García and de Lorenzo (2012). Plasmid DNA was obtained using the QIAprep Spin™ Miniprep kit (Qiagen, Inc., Valencia, CA, USA). Restriction enzymes were obtained from New England Biolabs Inc. (Ipswich, MA, USA), and T4 DNA ligase was purchased from Roche Applied Science Co. (Indianapolis, IN, USA). Plasmid p-R-SETA111 was constructed using isothermal assembly essentially as detailed by Gibson et al. (2009) but using a home-made mixture of enzymes. Colony PCR was performed using a single colony from a fresh LB agar plate and transferred directly into the reaction tube. PCR reactions were purified either with the NucleoSpin™ Gel and PCR clean-up kit (Macherey-Nagel GmbH & Co. KG, Düren, Germany) or the ExoSAP-IT™ PCR product clean-up kit (USB, Affymetrix Ltd., Santa Clara, CA, USA). Oligonucleotides were purchased from Sigma-Aldrich Co. (St. Louis, MO, USA). The oligonucleotides used in this work for specific DNA constructions are indicated in Table S1 in the Supplementary Material; the oligonucleotides used to identify the location of the chromosomal insertions are indicated in Table 2 (see also next section). The three different mini-Tn*5* modules were chemically synthesized *de novo* by GeneCust Europe S.A. (Dudelange, Luxembourg). DNA sequencing was carried out by Secugen SL (Madrid, Spain).

**Table 2:** **Oligonucleotides used in this work to identify and sequence the chromosomal locus of mini-Tn*5* insertions**.

Oligonucleotide	Sequence (5′ → 3′)	Use and reference
ARB6	GGCACGCGTCGACTAGTACNNNNNNNNNNACGCC	Arbitrary PCR, round 1 (Pratt and Kolter, 1998)
ARB2	GGCACGCGTCGACTAGTAC	Arbitrary PCR, round 2 (Pratt and Kolter, 1998)
pBAM-ME-I-Ext-R	CTCGTTTCACGCTGAATATGGCTC	Arbitrary PCR for pBAMD1-2, round 1 (Martínez-García et al., 2011)
pBAM-ME-I-Int-R	CAGTTTTATTGTTCATGATGATATA	Arbitrary PCR for pBAMD1-2, round 2, and sequencing (Martínez-García et al., 2011)
ME-O-Km-Ext-F	CGTCTGTTTCAGAAATATGGCAT	Arbitrary PCR for pBAMD1-2, round 1
ME-O-Km-Int-F	ATCTGATGCTGGATGAATTTTTC	Arbitrary PCR for pBAMD1-2, round 2, and sequencing
ME-I-Sm-Ext-R	ATGACGCCAACTACCTCTGATA	Arbitrary PCR for pBAMD1-4, round 1
ME-I-Sm-Int-R	TCACCGCTTCCCTCATGATGTT	Arbitrary PCR for pBAMD1-4, round 2, and sequencing
ME-O-Sm-Ext-F	CTTGGCCTCGCGCGCAGATCAG	Arbitrary PCR for pBAMD1-4, round 1
ME-O-Sm-Int-F	CACCAAGGTAGTCGGCAAAT	Arbitrary PCR for pBAMD1-4, round 2, and sequencing
ME-O-Gm-Ext-F	GCACTTTGATATCGACCCAAGT	Arbitrary PCR for pBAMD1-6, round 1
ME-O-Gm-Int-F	TCCCGGCCGCGGAGTTGTTCGG	Arbitrary PCR for pBAMD1-6, round 2, and sequencing
ME-I-Gm-Ext-R	GTTCTGGACCAGTTGCGTGAG	Arbitrary PCR for pBAMD1-6, round 1
ME-I-Gm-Int-R	GAACCGAACAGGCTTATGTCA	Arbitrary PCR for pBAMD1-6, round 2, and sequencing

Three separate DNA segments, carrying the transposon module along with the corresponding antibiotic-resistance determinant, were obtained as follows. In the first step, we used pBAM1 (Martínez-García et al., 2011) as the template to amplify the *bla* gene using primers Ap-*Asi*SI-F and Ap-*Mlu*I-R (Table S1 in the Supplementary Material), thereby substituting the *Swa*I and *Psh*AI restriction sites by target recognition sites for *Asi*SI and *Mlu*I, respectively, while maintaining the transcriptional control of *bla* through the native P3 promoter (Brosius et al., 1982). The backbone of plasmid pSEVA111 (Silva-Rocha et al., 2013) was amplified with the SEVA111-F and SEVA111-R oligonucleotide pair to obtain the second DNA fragment. Finally, the *tnpA* gene from pBAM1 was obtained using the oligonucleotides *tnpA*-*San*DI-F and *tnpA*-*Asi*SI-R that add the corresponding *San*DI and *Asi*SI restriction sites to the amplified fragment. These fragments were joined together by isothermal assembly, giving rise to plasmid p-R-SETA111 (Figure S1 in Supplementary Material).

Tripartite conjugative matings were set using *E*. *coli* CC118 λ*pir* (carrying pBAMD1-*x*, where *x* stands for any of the three antibiotic markers; see below) as the donor strain, the mating-helper strain *E*. *coli* HB101 (carrying pRK600), and *P*. *putida* KT2440 as the recipient strain. Conjugative matings were performed as described elsewhere (Martínez-García et al., 2011; Martínez-García and de Lorenzo, 2012). Briefly, the OD_600_ from overnight cultures grown in LB medium with the appropriate antibiotics was adjusted to 1, then the cells were washed twice with 10 mM MgSO_4_ to remove antibiotics from the culture medium, and each bacterial suspension was added to a 10 ml tube containing 5 ml of 10 mM MgSO_4_ to obtain a final OD_600_ of *ca*. 0.03. Biparental matings were done by following a similar procedure, but using *E*. *coli* S17-1 λ*pir* as the donor strain.

The mixture was concentrated by filtration and the cells were laid onto a filter disk (0.45 μm pore-size, 23 mm diameter, EMD Millipore Corp., Billerica, MA, USA). The filter was placed onto the surface of an LB agar plate and incubated at 30°C for 6 h. Finally, the biomass from the filter was suspended in 5 ml of 10 mM MgSO_4_ and different dilutions were plated onto a suitable selective media.

### Localization of the mini-Tn*5* transposon insertion sites by arbitrary PCR

In order to specifically map the landing sites of the mini-transposon within the chromosome, a set of oligonucleotides was designed that specifically hybridizes in each of the mini-Tn*5* elements (Table 2). Transconjugants were streaked onto M9 minimal medium agar plates with 0.2% (w/v) sodium citrate as the sole carbon source and supplemented with the appropriate antibiotics to obtain isolated colonies, which were re-streaked again in the same culture medium to obtain isolated clones. These colonies were used as the template for arbitrary PCR. The identification of the transposon insertion site could be independently obtained using either the oligonucleotides that are close to the ME-O end or with the ones designed for the ME-I end. However, when these plasmids carry any heterologous DNA in the multiple cloning site (MCS), the use of the oligonucleotides that are close to the ME-O end is the preferable option, since the ones for the ME-I end are located downstream the MCS and therefore would amplify through it. In the later case, and depending on the length of the DNA cloned in the MCS, the corresponding amplicon may not provide long enough a sequence to ascertain the insertion site of the mini-transposon module.

The conditions of the first round of arbitrary PCR were as follows: 5 min at 95°C (initial denaturation); six cycles of 30 s at 95°C, 30 s at 30°C, and 90 s at 72°C; and 30 cycles of 30 s at 95°C, 30 s at 45°C, and 90 s at 72°C (Das et al., 2005). The ARB6 oligonucleotide was used together with the external oligonucleotides within the mini-transposon (indicated with the “Ext” acronym in Table 2). Then, we used 1 μl of the first PCR round as the template for the second round of arbitrary PCR by applying the following conditions: 1 min at 95°C (initial denaturation); 30 cycles of 30 s at 95°C, 30 s at 52°C, and 90 s at 72°C; followed by an extra extension of 4 min at 72°C (Das et al., 2005). For the second round of arbitrary PCR, the ARB2 oligonucleotide was used together with the internal primers within the mini-transposon (indicated with the “Int” acronym in Table 2). Finally, the PCR amplification product obtained in the second round was directly purified and sent for sequencing with the corresponding internal oligonucleotide.

DNA sequences were thoroughly inspected visually for any error and analyzed using the *Pseudomonas* Genome Database (Winsor et al., 2011), and BlastN (Altschul et al., 1990, 1997) was subsequently employed to map the precise transposon insertion point. To ascertain the conservation level of the 9-bp target sequence of Tn*5* transposase, we resorted to the web-based application WebLogo 3.4 (Crooks et al., 2004).

### Analytical procedures

For a coarse estimation of the PHB content in *E*. *coli* transconjugants, we resorted to a fluorimetric assay based on Nile red staining (Spiekermann et al., 1999). Cells were grown overnight in LB medium with the proper antibiotic. The cultures were diluted to an OD_600_ of 0.1 in fresh LB medium containing 30 g l^−1^ glucose and 200 μl aliquots were placed in a 96 well microtiter plate (Costar™ black plates with clear bottom; Thermo Fisher Scientific Inc.). After growing the cells for 24 h at 37°C, 0.002 volume of a Nile red stock solution, freshly prepared by dissolving the dye (purchased from Sigma-Aldrich Co.) to 1 mg ml^−1^ in dimethyl sulfoxide, were added to each well. The microtiter plates were incubated at 37°C in the dark for 30 min, and the fluorescence at 585 nm was measured in a SpectraMax M2e plate reader (Molecular Devices, LLC., Sunnyvale, CA, USA) in cells before and after staining with Nile red. The raw fluorescence readings were normalized to the biomass in each well by dividing the values by the OD_600_ of the corresponding culture.

The quantification of PHB content in *E*. *coli via* fluorescence-activated cell sorting (FACS) was conducted by following a slight modification of the protocol described by Tyo et al. (2006). In brief, cultures to be analyzed were promptly cooled to 4°C by placing them in an ice bath for 15 min. Cells were harvested by centrifugation (5 min, 5,000 × *g*, 4°C), resuspended to an OD_600_ of 0.4 in cold TES buffer [10 mM Tris·HCl (pH = 7.5), 2.5 mM EDTA, and 10% (w/v) sucrose], and further incubated on ice for 15 min. Bacteria were recovered by centrifugation as explained above, and finally resuspended in the same volume of cold 1 mM MgCl_2_. A 1 ml aliquot of this suspension was added with 3 μl of an 1 mg ml^−1^ Nile red solution and incubated in the dark at 4°C for 30 min. Cells were analyzed by FACS immediately after the staining procedure. FACS was carried out in a MACSQuant™ VYB cytometer (Miltenyi Biotec GmbH, Bergisch Gladbach, Germany). Cells were excited with an Ar laser (488 nm, diode-pumped solid state), and the Nile red fluorescence at 585 nm was detected with a 614/50 nm band-pass filter. FACS analysis was done on at least 50,000 cells and the results were analyzed with the built-in MACSQuantify™ software 2.5 (Miltenyi Biotec). The geometric mean of fluorescence in each sample was correlated to the PHB content (expressed as a percentage) through a calibration curve as described previously (Tyo et al., 2006).

Cell-free extracts were obtained from cells harvested by centrifugation from an appropriate culture volume at 4,000 × *g* at 4°C for 10 min and processed as described previously (Nikel and de Lorenzo, 2013b; Nikel et al., 2014b). The total protein concentration in cell extracts was assessed by means of the Bradford method (Bradford, 1976) using a commercially available kit from BioRad Laboratories, Inc. (Hercules, CA, USA), with crystalline bovine serum albumin as the standard for determinations. *In vitro* quantification of the specific 3-ketoacyl-coenzyme A (CoA) thiolase activity in the thiolysis direction was conducted according to the protocols developed by Palmer et al. (1991) and Slater et al. (1998), with some modifications. The assay mixture (1 ml) contained 65 mM Tris–HCl (pH = 7.5), 50 mM MgCl_2_, 62.5 μM CoA, and 65 μM 3-acetoacetyl-CoA (Sigma-Aldrich Co.). Solutions of both CoA and 3-acetoacetyl-CoA were freshly prepared just prior to the assay. The assay was initiated upon the prompt addition of the cell-free extract, and the disappearance of 3-acetoacetyl-CoA was measured with time at 304 nm (using an extinction coefficient for 3-acetoacetyl-CoA ε_304_ = 16.9 × 10^3^ M^−1^ cm^−1^). One enzyme unit was defined as the amount of enzyme catalyzing the conversion of 1 μmol of substrate to product per min at 25°C.

Residual glucose and acetate concentrations in culture supernatants were determined in selected samples using adequate enzymatic kits (R-Biopharm AG, Darmstadt, Germany), essentially as per the manufacturer’s instructions. In either case, control mock assays were made by spiking M9 minimal medium with different amounts of the metabolite under examination. Metabolite yields and kinetic culture parameters were analytically calculated from the raw growth data as described elsewhere (Nikel et al., 2008a, 2010a, 2014b; Nikel and de Lorenzo, 2013a,b).

### Statistical analysis

The reported experiments were independently repeated at least twice (as indicated in the corresponding figure legend), and the mean value of the corresponding parameter ± SD is presented. When appropriate, data were statistically treated with an unpaired Student’s *t* test, and 95% confidence intervals for each parameter were calculated to demonstrate a statistically significant difference in means among the experimental samples. For the flow cytometry experiments, the geometric mean values (from which the PHB content is derived) were analyzed *via* the Mann–Whitney *U* test.

### Nucleotide sequence accession numbers

The sequences of the pBAMD vectors were deposited in the GenBank database with the following GenBank accession numbers: KM403113 (pBAMD1-2), KM403114 (pBAMD1-4), and KM403115 (pBAMD1-6).

## Results and Discussion

### Rationale, design, and general characteristics of the mini-Tn*5*-based vectors pBAMDs

Vector pBAM1 (*b*orn *a*gain *m*ini-transposon) is a synthetic and modular plasmid with a number of features to facilitate the genome editing of Gram-negative bacteria (Martínez-García et al., 2011). We decided to further extend the range of such applications by constructing a new set of pBAM1-derivative plasmids, which are compatible with the rules set in the *Standard European Vector Architecture* (SEVA) format (Silva-Rocha et al., 2013). We decided to accomplish this challenge by constructing a standardized version of the mini-transposon delivery plasmid that take full advantage of all the benefits of the pBAM1 plasmid together with the functional elements available within the SEVA collection. The starting idea was to design a plasmid series in which the mini-transposon module, the antibiotic-resistance marker, and the *tnpA* could be easily interchanged at the user’s will. In doing so, several changes were needed to re-structure the constituents of pBAM1, giving rise to three plasmids that were collectively termed pBAMD (i.e., *pBAM d*erivative) vectors. These insertion plasmids share all the advantages and several structural features of their pBAM1 predecessor. In brief, these features include (i) the narrow host-range origin of replication of plasmid R6K [*ori*(R6K)], dependent on the Π protein (encoded by the *pir* gene of plasmid R6K); (ii) an origin of transfer, *oriT*, that allows for the conjugative transfer of the plasmid from a host strain to a new bacterial recipient through RK2-mediated mobilization; (iii) the *bla*-encoded β-lactamase marker that confers resistance to ampicillin as a selective marker of the backbone vector; and [iv] a modified, hyper-active transposase encoded by *tnpA* just outside (but adjacent to) a DNA segment that is flanked by the terminal sequences of Tn*5* (i.e., the mini-transposon module itself). The Tn*5*-transposition system is an optimal source of biological parts because of its genetic promiscuity, and it can be considered to operate as a virtually orthogonal part, since it displays an autonomous behavior with respect to the host metabolic and regulatory traits.

We first constructed an intermediary plasmid, named p-R-SETA111, that was later used as the backbone in which the different mini-Tn*5* antibiotic modules were implanted to obtain the pBAMD1-*x* vectors. A mixture of three separate DNA fragments were isothermally assembled (Gibson et al., 2009) to construct the p-R-SETA111 intermediary plasmid (Figure S1 in Supplementary Material). The sequence of p-R-SETA111 was thoroughly checked after assembling with the set of oligonucleotides described in Table S1 in the Supplementary Material. This intermediate vector bears an R6K origin of replication that depends on the Π protein supplied in *trans* (Kolter et al., 1978) for replication. This situation calls for the use of *E*. *coli pir*^+^ strains to propagate these plasmids (Miller and Mekalanos, 1988; Herrero et al., 1990), such as *E*. *coli* DH5α λ*pir*, CC118 λ*pir*, or S17-1 λ*pir* (Table 1). Another feature of plasmid p-R-SETA111 is the presence of a minimized origin of transfer (*oriT*) from the promiscuous conjugative plasmid RP4 (Lyras and Rood, 1998; Silva-Rocha et al., 2013). Another trait of this intermediary vector shared with the SEVA plasmids is that the cargo module is flanked by the strong T1 and T0 transcriptional terminators (Silva-Rocha et al., 2013), which isolate transcriptionally any DNA sequence cloned in the MCS of p-R-SETA111. Importantly, the modular design of this plasmid allows for the convenient exchange of the *tnpA* by restriction of p-R-SETA111 with the rare cutters *San*DI (5′-GG/GWCCC-3′, W = A or T; Simcox et al., 1995) and *Asi*SI (5′-GCGAT/CGC-3′). Likewise, the antibiotic marker of the plasmid backbone (*bla* in this particular case), can be easily exchanged by enzymatic restriction with *Asi*SI and *Mlu*I (5′-A/CGCGT-3′).

Three different mini-Tn*5* modules were designed as the cargo segments to be implanted into the p-R-SETA111 plasmid. These elements were devised as cargoes for the SEVA plasmid collection (Silva-Rocha et al., 2013; Durante-Rodríguez et al., 2014), and so they were bracketed by *Pac*I and *Spe*I restriction sites. The cargo modules have a similar structural design, only differing in the antibiotic marker placed within the mini-transposon (see below). The mini-Tn*5* modules are flanked by the two mosaic end (ME) sequences. ME elements are optimized 19-bp DNA sequences recognized by the Tn*5* TnpA transposase to promote the specific transposition of any DNA segment bracketed by these elements (Zhou et al., 1998). Even though they are identical in sequence, and with the aim to facilitate the orientation of each functional element within the plasmid, we termed them as either ME-I (the one just after the *Pac*I recognition site) or ME-O (the one close to the *Spe*I recognition site).

The next relevant feature, placed right after the ME-I element, is a 10-bp buffer DNA sequence that is immediately followed by a MCS (spanning recognition sites for *Avr*II, *Sfi*I, *Not*I, *Eco*RI, *Sac*I, *Sma*I, *Bam*HI, *Xba*I, *Sal*I, *Pst*I, *Sph*I, *Hin*dIII, and *Not*I, in the 5′ → 3′direction). This DNA stretch has the same restriction sites as those present in cargo 1 in the SEVA database. The synthetic T500 terminator (Yarnell and Roberts, 1999) was placed after the MCS in order to avoid any transcriptional read-through that any heterologous DNA cloned within the cargo can leak into the following component of the plasmid. The next functional element of the mini-transposon is the antibiotic selection marker that allows for the proper selection of transconjugants. For this set of plasmids, we have used resistances to kanamycin (*aphA*, encoding an aminoglycoside 3′-phosphotransferase; Martínez-García et al., 2011), streptomycin/spectinomycin (*aadA*, encoding a streptomycin 3′(9)-*O*-nucleotidyl transferase; Fling et al., 1985), and gentamicin (*aacC1*, encoding a gentamicin 3′-*N*-acetyltransferase; Kovach et al., 1995). These antibiotic-resistance cassettes are coded as 2, 4, and 6 in the SEVA database. Since the antibiotic selection cassettes are flanked by *Swa*I and *Psh*AI recognition sites, the user has the ability to further expand the plasmid collection by exchanging the cognate resistance genes with any of the two other markers present in the SEVA collection, i.e., *cat* (encoding a chloramphenicol *O*-acetyltransferase) or *tetA* (encoding a tetracycline efflux protein). The *rho*-independent transcriptional terminator from the gene 32 encoded in the phage T4 genome (Gorski et al., 1985; Miller et al., 2003; Martínez-García et al., 2011) was placed immediately downstream to the antibiotic-resistance gene to prevent any possible read-through from the corresponding promoter elements. Furthermore, the motif 5′-**G**GGACCC-3′ was changed to 5′-**C**GGACCC-3′ to eliminate a *San*DI restriction target within the existing terminator sequence.

**Figure 1:**
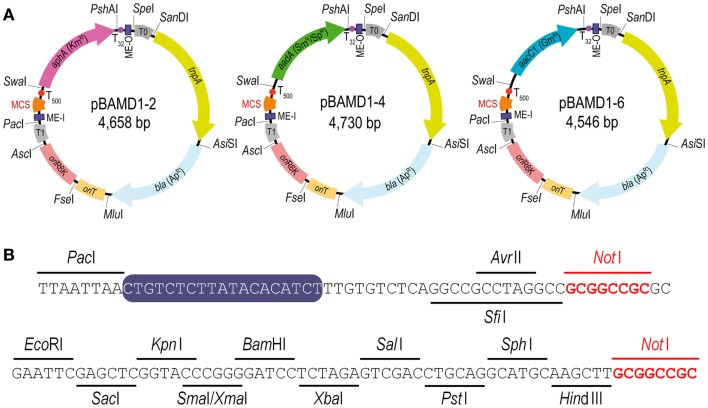
**Functional features of the pBAMD1-*x* delivery vectors**. **(A)** Schematic representation of the pBAMD1-*x* plasmid series. The functional elements of each plasmid include the relevant restriction sites used for assembling the vectors, the antibiotic-resistance markers (Ap, ampicillin; Km, kanamycin; Sm, streptomycin; Sp, spectinomycin; and Gm, gentamicin), the hyper-active TnpA transposase encoded by *tnpA*, a conditional origin of replication [*ori*(R6K)] dependent of the π protein, an origin of transfer (*oriT*), two mosaic elements (termed ME-I and ME-O), the transcriptional terminators T0 and T1 located just outside the transposon module, and a multiple cloning site (MCS) compatible with any plasmid belonging to the *Standard European Vector Architecture* (SEVA) initiative (Silva-Rocha et al., 2013; Durante-Rodríguez et al., 2014). Note that the antibiotic-resistance gene determines the full name of each plasmid. **(B)** Restriction enzymes targets within the multiple cloning site of the pBAMD1-*x* delivery vectors (*x* = 2, Km^R^; *x* = 4, Sm^R^/Sp^R^; and *x* = 6, Gm^R^). The MCS starts with the unique *Avr*II/*Sfi*I recognition sites, and two *Not*I recognition sites (highlighted in red) were included in the MCS sequence to enable the consecutive assembly of different cargos from the SEVA collection. The sequence of ME-I is indicated by a purple box.

The last step in the construction of the pBAMD1-*x* vectors was the insertion of the mini-transposon modules themselves into the p-R-SETA111 backbone. The sequences of the three mini-Tn5 modules were edited *in silico* to follow the SEVA rules and synthesized *de novo*. The mini-transposon modules were restricted using *Pac*I and *Spe*I and inserted into p-R-SETA111 previously digested with the same enzymes. The correctness of this last cloning step was verified by DNA sequencing with the SEVA oligonucleotides PS1 and PS2 (Table S1 in the Supplementary Material), that flank the cargo module. The resulting delivery plasmids were termed pBAMD1-2 (Km^R^), pBAMD1-4 (Sm^R^/Sp^R^), and pBAMD1-6 (Gm^R^) (Figure 1A). Note that the first digit numeric nomenclature stems for the fact that all the pBAMD1-*x* vectors are derivatives of the pSEVA111 vector, while the second number identify the antibiotic-resistance marker. All the vectors share the same MCS (Figure 1B), also compatible with the rest of SEVA vectors already available.

### Functional validation of the pBAMD1-*x* vectors in *P*. *putida* KT2440

To evaluate the functionality of the new set of Tn*5* plasmids, the frequency of transposition into the environmental bacterium *P*. *putida* KT2440 was firstly tested in 6 h triparental mating assays. In order to estimate the frequency of transposition events, the number of antibiotic-resistant colonies was assessed after 24 h of incubation at 30°C, and normalized to the total of 1.5 × 10^8^ recipient cells used in each experiment. The average frequency of transposition obtained at 6 h was (3.8 ± 2.9) × 10^−4^ transconjugants (ranging from a minimum of 2.5 × 10^−5^ to a maximum of 1.0 × 10^−3^ transconjugant cells). These figures were independent of the antibiotic marker used (either pBAMD1-2, pBAMD1-4, or pBAMD1-6), and no spontaneous antibiotic-resistant clones (i.e., Km^R^, Sm^R^, or Gm^R^, respectively) were detected under such growth conditions (data not shown).

The next step was to differentiate between *bona fide* transposition events and non-specific plasmid integration events in the *P*. *putida* genome. To do so, transconjugants cells were re-streaked onto LB medium plates containing 500 μg ml^−1^ ampicillin and incubated for 24 h to check for possible growth – which would indicate that the corresponding pBAMD vector had integrated into the target chromosome instead of transposing the MEs-flanked DNA cargo. Besides this simple test, the user could also perform colony PCR amplifications to confirm that the transconjugants do not have the pBAMD plasmid backbone by using any of the following two SEVA oligonucleotides combination. If the plasmid is present, the PS5-PS4 oligonucleotide pair will produce a 225-bp amplicon within the *oriT* region, and the PS5-PS6 oligonucleotide pair will generate a 665-bp amplicon including the *oriT* segment and the R6K origin of replication (Silva-Rocha et al., 2013). By conducting these two assays, we noticed that 4.2 ± 2.4% of the potential transconjugants obtained with the pBAMD vectors resulted from plasmid co-integration events (a percentage very similar to that reported for other Tn*5*-based plasmid systems; de Lorenzo et al., 1990). Therefore, it is highly recommended to confirm the nature of the antibiotic-resistant clones obtained after each round of insertions.

**Figure 2:**
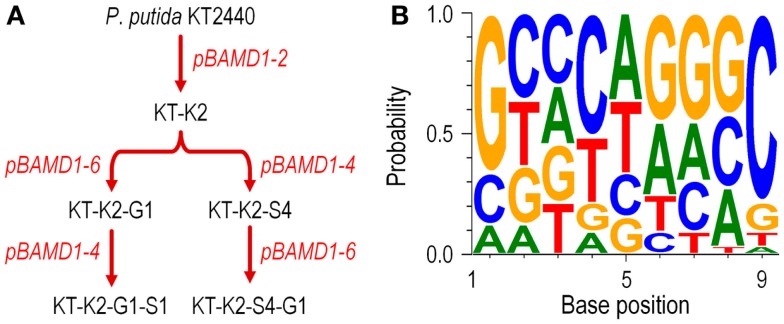
**Functional characterization of the pBAMD1-*x* delivery vectors in *P*. *putida* KT2440**. **(A)** Sequential insertion of different mini-Tn*5* modules from pBAMD1-*x* plasmids carrying all the three possible antibiotic-resistance determinants. The flowchart shows the procedure followed for the combinatorial integrations, starting from the wild-type strain KT2440. The names given to the intermediate strains reflect the order in which each antibiotic was delivered into the recipient bacteria (K, kanamycin; S, streptomycin/spectinomycin; and G, gentamicin). The exact chromosomal localization of the insertions in these strains is given in Table 3. Plasmids used in each round of integration are indicated in red. **(B)** Assessment of the possible sequence preference in the target DNA during the insertion process of the pBAMD1-*x* delivery vectors in *P*. *putida* KT2440. The WebLogo 3.4 software was used to identify the DNA signature (if any) in which the transposon lands in the chromosome of recipient bacteria. The software was fed with the 9-bp DNA sequence targeted by mini-Tn*5* in independent trials (Table 3). Note the slight preference for G/C pairs at both ends of the target DNA motif (i.e., in positions 1 and 9).

We also studied whether the pBAMD mini-transposon delivery plasmids can be used serially to generate double and even triple transconjugant mutants by taking advantage of the three different antibiotic resistance markers (Figure 2A). A first round of transposition was performed with the three individual pBAMD plasmids using *P*. *putida* KT2440 as the recipient strain. Transconjugants were re-streaked onto M9 minimal medium plates containing 0.2% (w/v) citrate and supplemented with the appropriate antibiotics to obtain isolated colonies. These colonies were re-streaked again in the same culture medium to obtain pure clones. We randomly picked 22 clones obtained with each pBAMD1-*x* plasmid and characterized the corresponding transposon insertion site. In 12 out of 22 transconjugant clones, the ME-O related oligonucleotides (Table 2) were used, while in the remaining 10 transconjugant clones, the sequence was obtained by using the ME-I related oligonucleotides for the arbitrary PCR amplification. One Km^R^ clone after the first round of transposition with pBAMD1-2 was selected and used as the recipient strain for the pBAMD1-4 and pBAMD1-6 mini-transposon plasmids. After this second insertion round, we selected four transconjugants obtained with each system, and mapped the landing point of the mini-transposon using only the ME-O related oligonucleotides. Finally, we selected one *P*. *putida* KT2440 Km^R^ and Gm^R^ and one *P*. *putida* KT2440 Km^R^ and Sm^R^ to perform the third round of transposition with either pBAMD1-4 or pBAMD1-6, respectively. In the last step, we have chosen one mutant per plasmid system and characterized again the insertion site of the three mini-transposons using the ME-O oligonucleotides. The precise site of transposon insertion could be ascertained in 30 transconjugants out of 32 independent clones (Table 3). Specifically, in one of the cases in which it was not possible to identify the localization of the mini-Tn*5* element, the transposon insertion could have happened in either PP2612 or PP3616, since both genes share a 97% sequence identity. In the other case, the transposon insertion site could not be precisely mapped due to the presence of a large number of internal repeats within the *lapA* gene (PP0168).

**Table 3:** **Insertion sites of the mini-transposon born by the different pBAMD1-*x* vectors in the genome of *P*. *putida* KT2440*^a^***.

Clone	End	Genome coordinate (bp)	PP number	Strand	Gene name and putative function
KT-K1	ME-I	5,825,403	PP5103	−	*trmB*, tRNA (guanine-*N*_7_-) methyltransferase
KT-K2	ME-I	425,161	PP0349	−	PepSY-associated transmembrane helix domain-containing protein
KT-K3	ME-I	5,272,887	PP4648	+	rRNA (guanine-*N*_2_-) methyltransferase
KT-K5	ME-O	864,889	PP0745	−	*uraA*, uracil-xanthine permease
KT-K6	ME-O	4,661,528	PP4124	+	*nuoG*, NADH dehydrogenase subunit G
KT-K7	ME-O	1,261,995	PP1104	−	Succinyl-glutamate desuccinylase/aspartoacylase
KT-K8	ME-O	727,311	PP0621	−	Hypothetical protein
KT-G1*^b^*	ME-O	2,988,216	PP2612	−	Hypothetical protein
		4,108,354	PP3616	+	Hypothetical protein
KT-G2	ME-O	1,313,801	PP1145	−	*hepA*, ATP-dependent helicase
KT-G3	ME-O	3,002,652	PP2626	+	Hypothetical protein
KT-G4	ME-O	3,251,143	PP2847	+	*ureJ*, hydrogenase/urease accessory protein
KT-G6	ME-I	4,444,716	PP3941	−	Isochorismatase superfamily hydrolase
KT-G7	ME-I	4,792,981	PP4221	−	Non-ribosomal peptide synthetase
KT-G8	ME-I	1,959,862	PP1757	+	*bolA*, BolA family protein
KT-S1	ME-I	4,463,594	PP3956	+	Hypothetical protein
KT-S2	ME-I	3,552,337	PP3136	NA	Intergenic region (PP3136 and PP3137)
KT-S3	ME-I	4,671,241	PP4132	−	Hypothetical protein
KT-S4	ME-I	35,484	PP0031	−	Hypothetical protein
KT-S5	ME-O	2,422,455	PP2123	+	*moeA*, molybdopterin biosynthesis enzyme
KT-S6	ME-O	6,021,399	PP5272	−	Hypothetical protein
KT-S7	ME-O	381,423	PP0317	−	Methyl-accepting chemotaxis sensory transducer
KT-S8	ME-O	328,477	PP0270	−	Integral membrane sensor signal transduction histidine kinase
KT-K2-G1	ME-O	4,892,623	PP4301	+	*pykF*, pyruvate kinase
KT-K2-G2	ME-O	3,749,626	PP3313	−	Heat shock protein
KT-K2-G3	ME-O	5,842,545	PP5120	+	Conifer aldehyde dehydrogenase
KT-K2-G4	ME-O	1,373,720	PP1199	−	Hypothetical protein
KT-K2-S1	ME-O	2,904,080	PP2556	−	Chromate transporter
KT-K2-S2	ME-O	5,347,381	PP4703	+	Hypothetical protein
KT-K2-S3	ME-O	1,542,387	PP1353	+	Mechanosensitive ion channel protein MscS
KT-K2-S4	ME-O	5,892,275	PP5166	−	Fis family transcriptional regulator
KT-K2-G1-S1	ME-O	3,635,742	PP3202	+	Predicted exporter of the RND superfamily
KT-K2-S4-G1*^b^*	ME-O	NA	PP0168	+	*lapA*, surface adhesion protein

*^a^The insertion sites were ascertained by means of arbitrary PCR with the oligonucleotides indicated in Table 2, and the assigned function of the corresponding ORF is given according to the information available in the *Pseudomonas* Genome Database (Winsor et al., 2011). NA, not available*.

*^b^In the two transconjugant clones indicated, the insertion site of the mini-Tn*5* element could not be unambiguously identified*.

We then used the insertion sequence data of each mapped clone in Table 3 to detect any sequence preference for the integration of the mini-Tn*5* cassettes within the genome of *P*. *putida* KT2440. The web-based application WebLogo 3.4 (Crooks et al., 2004) was fed with the 9-bp landing sequence targeted by the mini-transposon. The program was set to show the probability of having a defined base at any specific position within the 9-bp motif, and the G + C percentage of the genome was adjusted to the value of *P*. *putida* KT2440 (G + C = 61.5%; Nelson et al., 2002). The results shown in Figure 2B reveal that there is no DNA sequence bias for the integration site of the mini-transposon, in a similar fashion as observed for plasmid pBAM1 (Martínez-García et al., 2011). However, a relatively minor preference for G/C pairs at both ends of the target DNA motif could be observed, as detected for other systems based on Tn*5* (Lodge et al., 1988). These experiments confirm that the three mini-transposons borne by the pBAMD1-*x* vectors could be used serially to generate a second or even a third round of insertion mutagenesis procedure, or also to stably integrate multiple genetic devices into the genome of a single microbial strain.

### Design and construction of a microbial cell factory for poly(3-hydroxybutyrate) synthesis

#### Engineering an stable PHB^+^ phenotype in *E*. *coli*

**Figure 3:**
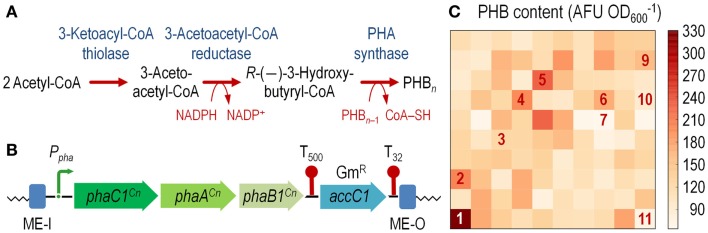
**Construction of an *E*. *coli* cell factory expressing the *phaC1AB1* gene cluster from *C*. *necator* as a chromosomal insertion**. **(A)** Poly(3-hydroxybutyrate) (PHB) biosynthesis pathway. Three enzymes are necessary for *de novo* synthesis of PHB in *C*. *necator*: a 3-ketoacyl-coenzyme A (CoA) thiolase (PhaA), a NADPH-dependent 3-acetoacetyl-CoA reductase (PhaB1), and a PHB synthase (PhaC1). PhaA and PhaB1 catalyze the condensation of two molecules of acetyl-CoA to 3-acetoacetyl-CoA and the reduction of acetoacetyl-CoA to *R*-(–)-3-hydroxybutyryl-CoA, respectively. PhaC1 polymerizes these monomers to PHB, whereas one CoA-SH molecule per monomer is released. The resulting PHB polymer is stored as water-insoluble granules in the cytoplasm of the cells. **(B)** Organization of the functional elements borne by plasmid pBAM1-6-*pha* and transferred into the chromosome of the recipient *E*. *coli* strain. The transcriptional terminators included in the plasmid backbone, which flank the gentamicin resistance (Gm^R^) determinant (*accC1*), are depicted as T_500_ and T_32_. Note that the elements in this outline are not drawn to scale. **(C)** Exploring the landscape of PHB synthesis in *E*. *coli* transconjugants. The *phaC1AB1* gene cluster from *C*. *necator* was randomly integrated into the chromosome of *E*. *coli* JW2293-1 (Δ*pta*), and 24-h cultures of individual colonies were analyzed for PHB accumulation by fluorimetry after staining the cells with Nile red (see Materials and Methods for details). Several colonies, identified by numbers in the heat-map, were kept and further analyzed to establish the precise site of mini-Tn*5*(*phaC1AB1*) insertion (Table S2 in the Supplementary Material). AFU, arbitrary fluorescence units.

Poly(3-hydroxybutyrate) is an isotactic polyester composed by 3-hydroxybutyrate units (Anderson and Dawes, 1990). The PHB synthesis pathway in *C*. *necator* (formerly known as *Ralstonia eutropha*) comprises three enzymes (Figure 3A) (Steinbüchel and Hein, 2001). PhaA, a 3-ketoacyl-CoA thiolase, condenses two acetyl-CoA moities, yielding 3-acetoacetyl-CoA. This intermediate is the substrate for PhaB, a NADPH-dependent 3-acetoacetyl-CoA reductase (encoded by *phaB1*). In the final step of this biosynthetic pathway, (*R*)-(–)-3-hydroxybutyryl-CoA is polymerized to PHB by PhaC, a poly(3-hydroxyalkanoate) synthase (encoded by *phaC1*). The very idea of a thermoplastic and biocompatible material, which is also readily biodegraded by a number of bacteria has become very attractive in an era of increasing environmental concern (Keshavarz and Roy, 2010). A number of different recombinant *E*. *coli* strains designed for polymer accumulation have been constructed thus far (Li et al., 2007; Chen et al., 2013; Ruiz et al., 2013), outsourcing the *pha* genes from several bacteria (Verlinden et al., 2007). However, most of the PHB production systems available thus far suffer from a number of drawbacks (Wang et al., 2014). Among them, the transcriptional regulation of the *pha* genes is of particular importance. In natural producer bacteria, PHB accumulation is triggered by an imbalance in the availability of critical nutrients (e.g., the N or S source; Anderson and Dawes, 1990). In recombinant *E*. *coli*, however, the constitutive expression of the *pha* genes leads to a growth-dependent accumulation of PHB, which normally results in metabolic burden in the producing cells (Wang and Lee, 1997). Controlling the rate of polymer accumulation in recombinant *E*. *coli* is thus of paramount importance for the design of efficient microbial cell factories. Besides this feature, the segregational stability of plasmids in *E*. *coli* recombinants could also be an issue in prolonged fermentation processes aimed at biopolymer production (Nikel et al., 2010b; Ruiz et al., 2013).

To overcome this state of affairs, we decided to explore the landscape of potentially useful transcription levels in *E*. *coli* by randomly integrating the *pha* genes from *C*. *necator* into the chromosome. To this end, we used vector pBAMD1-6 as the backbone to clone a 5.3-kb DNA fragment spanning the *phaC1AB1* genes. Plasmid pAeT41 (Peoples and Sinskey, 1989) was digested with *Eco*RI and *Sma*I to liberate the aforementioned DNA segment, and inserted into the corresponding restriction sites of pBAMD1-6 to generate plasmid pBAMD1-6-*pha* (Table 1). The functional parts of the DNA element to be transferred into *E*. *coli* are shown in Figure 3B.

As acetyl-CoA is the precursor metabolite for PHB formation, competing pathways that use this intermediate are expected to drain building blocks of the biopolymer synthesis. In *E*. *coli*, the acetate formation pathway, comprising Pta and AckA (phosphotransacetylase and acetate kinase), uses acetyl-CoA as the starting metabolite (Neidhardt et al., 1990). This pathway, which diverts a considerable amount of carbon from the central metabolism (Wolfe, 2005), is active under both oxic and anoxic conditions (Clark, 1989). For this reason, the *phaBAC* gene cluster was delivered into a Δ*pta* recipient strain (Table 1), in which the acetate formation is expected to be low. Plasmid pBAMD1-6-*pha* was first transferred to *E*. *coli* S17-1 λ*pir* to perform a biparental mating. *E*. *coli* S17-1 λ*pir* or SM10 λ*pir* are the preferred donor strains when mobilizing RP4-based plasmid to *E. coli* recipient strains since they bear the functional *tra* and *mob* elements integrated in the genome, thus avoiding the inadvertent transfer of the mating-helper plasmid (pRK600) alongside the mini-transposon delivery system. Alternatively, *E*. *coli* strain MFD *pir*^+^ (Ferrières et al., 2010), devoid of the Mu element present in the strains detailed above, could be used for transposon insertions.

After integration of the mini-Tn*5*:*phaC1AB1* device into *E*. *coli* JW2293-1, individual Gm^R^ colonies were purified and separately grown in microtiter plates as explained in the Section “Materials and Methods” to explore PHB accumulation in 100 independent transconjugants after 24 h of incubation. Figure 3C shows the level of PHB accumulation in the transconjugants as compared to that of *E*. *coli* JW2293-1 carrying the pAeT41 plasmid, in which the expression of the *phaC1AB1* gene cluster is driven by the native promoter. All the transconjugants tested accumulated the polymer from mono-copy chromosomal insertions of the PHB biosynthesis pathway to some extent, ranging from 3% up to 78% of the accumulation levels observed in *E*. *coli* JW2293P (which carries plasmid pAeT41). Table S2 in the Supplementary Material shows the insertion locus for some selected *phaC1AB1*^+^ transconjugants, clearly illustrating the non-selective nature of the chromosomal incorporation of Tn*5* (and consequently, the wide range of PHB accumulation levels), as it was already observed in *P*. *putida* transconjugants. We selected one of the transconjugants, termed *E*. *coli* TA2293P, that accumulated high polymer levels (clone 1 in Figure 3C and Table S2 in Supplementary Material), and the insertion site of the transposon was determined to be *ykgH*, an open reading frame encoding a predicted inner membrane protein (Table S2 in the Supplementary Material). The physiology of PHB accumulation of this strain was studied as detailed below.

#### Physiological and biochemical characterization of *E*. *coli* TA2293P as a microbial cell factory for PHB synthesis

The growth parameters of several *E*. *coli* strains were determined to explore their potential as biopolymer cell factories (Table 4). We decided to compare the performance of strains in which the *pha* genes are expressed either in a multi-copy plasmid or as a mono-copy insertion in the bacterial chromosome side-by-side. Interestingly, the elimination of Pta resulted in a reduction of the specific growth rate, probably by an imbalance in the acetyl-CoA pool that was partially restored by the heterologous expression of the *phaC1AB1* gene cluster. This positive effect was more evident in the strain in which the genes were inserted into the chromosome (the specific growth rate attained *ca*. 85% of that in the wild-type strain), thus suggesting that the adequate expression level of the PHB biosynthesis genes is important to recover a homeostatic acetyl-CoA balance. This kinetic pattern was also mirrored in the final biomass density of the cultures. In fact, among the strains tested, *E*. *coli* TA2293P attained the highest cell density. This result highlights the advantage of integrating the *pha* genes in the chromosome, as such approach not only avoids the metabolic burden usually associated with heterologous gene expression from plasmids and other extra-chromosomal elements but it also allows for the selection of an integrant strain exhibiting the appropriate level of transcription of the corresponding genes. Moreover, the selection of *ykgH* as a target was not at all obvious, indicating, again, the value of random insertion of the genes of interest in the bacterial chromosome.

**Table 4:** **Physiological characterization of wild-type and mutant *E*. *coli* strains as microbial cell factories for PHB biosynthesis in shaken-flask cultures**.

*E*. *coli* strain	Relevant characteristics	Physiological parameter*^a^*		
		μ (h^−1^)	CDW (g l^−1^)	*Y*_A/S_ (mol mol^−1^)
BW25113	Wild-type strain	0.74 ± 0.04	3.8 ± 0.2	0.64 ± 0.05
JW2293-1	Δ*pta*	0.44 ± 0.03	2.9 ± 0.3	0.13 ± 0.02
JW2293P	Δ*pta phaC1AB1*^+^	0.51 ± 0.07	3.5 ± 0.1	0.05 ± 0.02
TA2293P	Δ*pta ykgH*:mini-Tn*5*(*phaC1AB1*)	0.63 ± 0.05	4.2 ± 0.4	0.09 ± 0.03

*^a^Cells were grown aerobically in M9 minimal medium containing 30 g l^−1^ glucose as the sole carbon source. The specific growth rate (microns) was determined during logarithmic growth, whereas the final cell density (expressed as the cell dry weight, CDW) and the molar yield of acetate on glucose (Y_A/S_) were calculated after 24 h of incubation. Reported results represent the mean value* ± *SD of triplicate measurements from at least two independent cultures*.

Since we selected a *pta* mutant of *E*. *coli* as the recipient strain, in which acetate formation is expected to be impaired, by-product formation was also explored in these cultures as a measure of the carbon flow from glucose to PHB (Table 4). In cultures of the wild-type strain, up to 60% of the total carbon source was converted into acetate, pinpointing this metabolite as the key by-product of hexose catabolism in *E*. *coli* (and therefore, as the main side pathway competing for acetyl-CoA). The *pta* mutant still produced some acetate (probably through the action of pyruvate oxidase, PoxB); however, the molar conversion of glucose into acetate reached only *ca*. 20% of that observed in the wild-type strain. Acetate formation in both *E*. *coli* strains carrying PhaC1AB1 was comparable, and much lower than the other two strains. In all, these results bear witness of (i) the suitability of a *pta* mutant, deficient in acetate formation, as the starting point to construct a PHB cell factory, and (ii) the effect of the PHB biosynthetic pathway in using acetyl-CoA as the precursor metabolite. Once the coarse physiological characterization of these strains was completed, the next relevant question was how they perform as PHB producers.

**Figure 4:**
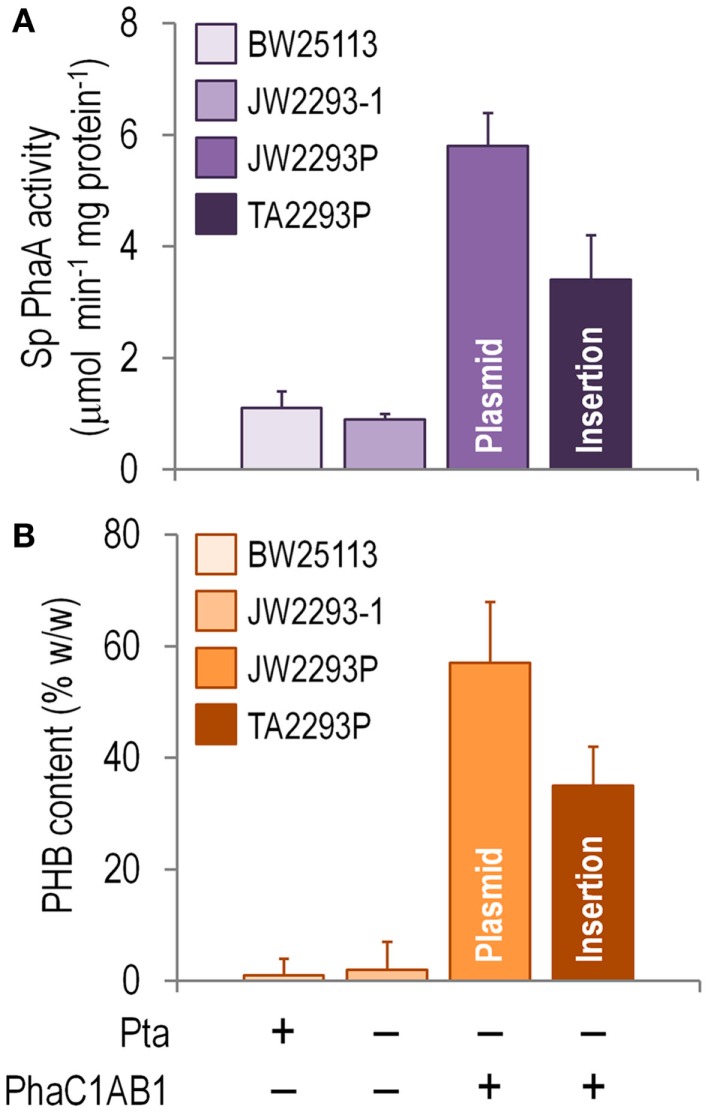
**Biochemical characterization of *E*. *coli* TA2293P as a microbial cell factory for PHB synthesis**. **(A)** *In vitro* determination of the specific (Sp) 3-ketoacyl-coenzyme A thiolase (PhaA) activity. Cells were harvested after growing them for 24 h in M9 minimal medium added with 30 g l^−1^ glucose as the sole carbon source, and the activity of PhaA was determined in the cell-free extract as detailed in the Section “Materials and Methods.” **(B)** Poly(3-hydroxybutyrate) (PHB) accumulation. The PHB content (expressed as a percentage of the cell dry weight) was assessed by flow cytometry after growing the cells for 24 h in M9 minimal medium added with 30 g l^−1^ glucose as the sole carbon source. In all cases, each bar represents the mean value of the corresponding enzymatic activity ± SD of triplicate measurements from at least two independent experiments. The strains used to explore these biochemical traits were *E*. *coli* BW25113 (wild-type strain), *E*. *coli* JW2293-1 (Δ*pta*), *E*. *coli* JW2293P (Δ*pta*, carrying the *phaC1AB1* gene cluster in a multi-copy plasmid), and *E*. *coli* TA2293P [Δ*pta, ykgH*:mini-Tn*5*(*phaC1AB1*)]. See Table 1 for further details about the genotype of each *E*. *coli* strain. The relevant features of each strain are indicated at the bottom of the figure.

Since PhaA is the first committed enzymatic step of PHB formation from acetyl-CoA, the *in vitro* activity of this enzyme was assayed as a proxy of the activity of the whole biosynthetic pathway in shaken-flask cultures using glucose as the sole carbon source (Figure 4A). Note that there was some degree of thiolase activity in both *E*. *coli* BW25113 and JW2293-1, probably represented by FadA (an enzyme normally involved in the degradation of fatty acids via the β-oxidation cycle). However, the specific PhaA activity was six and fourfold higher in the strains carrying the *phaC1AB1* gene cluster (*E*. *coli* JW2293P and TA2293P, respectively) as compared to that of *E*. *coli* JW2293-1. As expected, the highest enzymatic activity corresponded to the strain carrying the *pha* genes in a multi-copy plasmid. Nevertheless, *E*. *coli* TA2293P had an activity level *ca*. 60% of the strain bearing pAeT41, indicating that the appropriate insertion of the gene cluster could result in thiolase activities similar to those of a typical recombinant PHB producer. Yet, how do these activities translate into PHB accumulation?

Figure 4B shows that *E*. *coli* TA2293P accumulated PHB up to 62% of the level observed in the same strain but expressing the *pha* genes in a plasmid (i.e., *E*. *coli* JW2293P). As previously observed in other traits during the physiological characterization, the later strain had the highest PHB accumulation level among all the strains tested. That *E*. *coli* TA2293P accumulates such a high amount of PHB is a somewhat surprising (and welcome) result considering the difference in copy number between the two *phaC1AB1*^+^ strains under comparison. Note that a possible effect of the absence (or an altered expression level) of YkgH on the properties of *E*. *coli* TA2293P cannot be completely ruled out. Interestingly, when this strain was persistently cultured in LB medium without any selective pressure, its phenotypic traits, particularly regarding polymer accumulation, remained unchanged. By contrast, the segregational stability of pAeT41 was assessed in cultures of *E*. *coli* JW2293P, and, after growing the cells in LB medium and sub-culturing them daily seven times without any antibiotic, <25% of the cells were resistant to ampicillin.

## Conclusion

In our current study, we presented a set of new mini-Tn*5*-derived vectors that can be used to engineer the genome of Gram-negative bacteria. While the worth of these tools has been exposed in two model bacteria, *P*. *putida* and *E*. *coli*, the inherent promiscuity of Tn*5* ensures its functioning in a number of different microbial hosts. Of particular importance is the possibility of sequentially using the three pBAMD1-*x* vectors, thereby enabling the user to accumulate insertions in the same genetic background in a combinatorial fashion. This is a particularly interesting feature for the construction of complex phenotypes, such as biopolymer formation, which depend on more than one enzyme. Although the expression of the biosynthetic genes in a multi-copy plasmid is in principle enough to bestow the desired phenotype on the recipient bacterium, fine-tuned expression levels (together with the appearance of emergent phenotypic properties in the host, brought about by the insertion process itself) can be easily achieved by randomly integrating the structural genes into the chromosome. In this way, and since the genetic context of each integration will surely result in different regulatory patterns at the transcriptional level, the user could choose among a library of insertions those clones that meet any desired criterion (in our case, PHB accumulation). Moreover, as the delivery plasmids described in this study can be used in a sequential manner, other polymer-associated enzymes, such as phasins, can also be incorporated in the same strain to enhance further polymer production.

## Conflict of Interest Statement

The authors declare that the research was conducted in the absence of any commercial or financial relationships that could be construed as a potential conflict of interest.

## Supplementary Material

The Supplementary Material for this article can be found online at http://www.frontiersin.org/Journal/10.3389/fbioe.2014.00046/abstract
