# Supplementary material for: New Transposon Tools Tailored for Metabolic Engineering of Gram-Negative Microbial Cell Factories
Source: Front Bioeng Biotechnol. 2014 Oct 28;2:46. doi: 10.3389/fbioe.2014.00046 (PMC4211546; doi:10.3389/fbioe.2014.00046)
Supplement: Supplementary file 1 [file Presentation1.PDF]

Supplementary material for

New transposon tools tailored for metabolic engineering of Gram-negative microbial cell factories

by

Esteban Martínez-García, Tomás Aparicio, Víctor de Lorenzo, and Pablo I. Nikel

Fig. S1. Flowchart followed for the construction of vector p-R-SETA111.

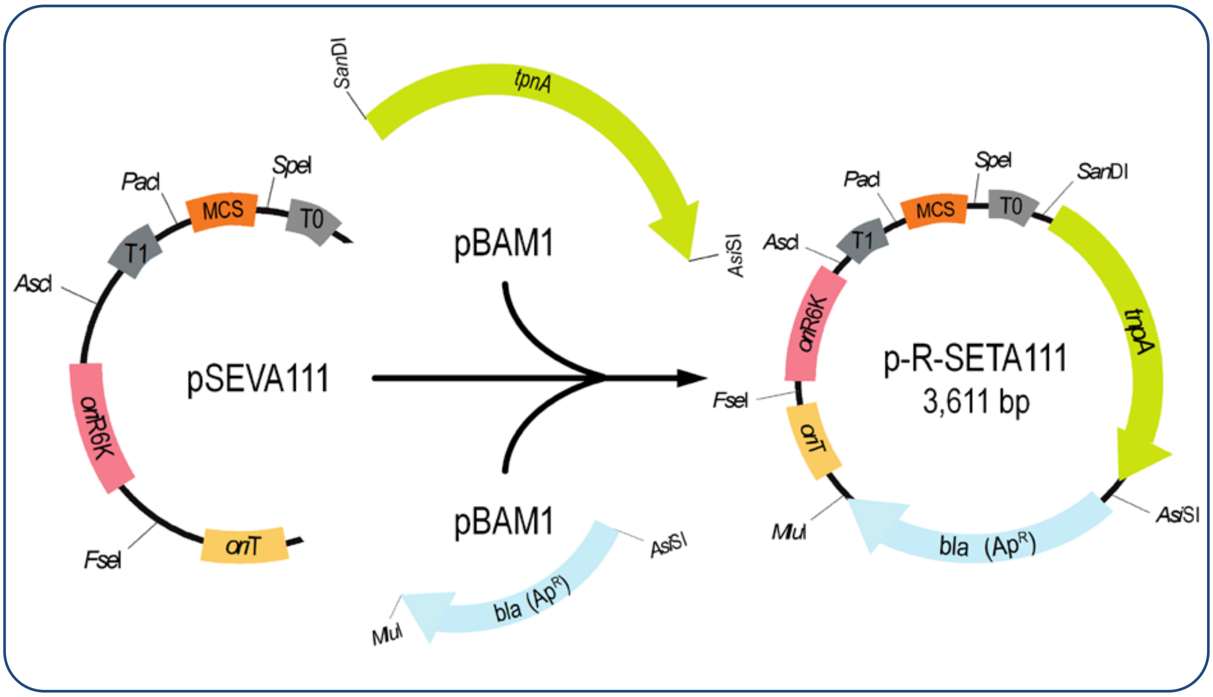

In the first step, the pBAM1 vector (Martínez-García et al., 2011) was used as the template to amplify the *bla* gene (conferring resistance to ampicillin) using primers Ap-*Asi*SI-F and Ap-*Mlu*I-R (see Table S1 for sequence details), which substitute the original *Swa*I and *Psh*AI restriction sites by target recognition sites for *Asi*SI and *Mlu*I, respectively. The transcriptional control of *bla* through the native P3 promoter (Brosius et al., 1982) was left intact. The DNA backbone of plasmid pSEVA111 (Silva-Rocha et al., 2013) was amplified by PCR with the SEVA111-F and SEVA111-R oligonucleotide pair to obtain the second DNA fragment. Finally, the *tnpA* gene from pBAM1 was obtained using the oligonucleotides *tnpA*-*San*DI-F and *tnpA*-*Asi*SI-R, that add the corresponding *San*DI and *Asi*SI restriction sites to the amplified fragment. These fragments were joined together by isothermal assembly as described by Gibson et al. (2009).

Table S1. Oligonucleotides used in this study.

Oligonucleotide	Sequence (5' → 3')[a]	Use and reference
tnpA-*San*DI-F	GCCGCCGGGCGTTTTTTATTGGTGAGAATC<br>CAG**GGGTCCC**CTGGTTTAAACTACACAAG	Amplification of *tnpA*
tnpA-*Asi*SI-R	CTCATTAGGCGGGCTACTA**GCGATCGC**CGA<br>TTAGATTTTAATGCCC	Amplification of *tnpA*
Ap-*Asi*SI-F	GGGCATTAAAATCTAATCG**GCGATCGC**TAG<br>TAGCCCGCCTAATGAG	Amplification of *bla*
Ap-*Mlu*I-R	TAATGACCCCGAAGCAGGGTTATGCAGCGG<br>AAAAGGACAA**ACGCGT**GTCCAAAAAAAAAG<br>GCTCC	Amplification of *bla*
SEVA111-F	TTGTCCTTTTCCGCTGCATAAC	Amplification of the pSEVA111 plasmid backbone
SEVA111-R	GACCCCTGGATTCTCACC	Amplification of the pSEVA111 plasmid backbone
tnpA-Check-F	ATGCGGATGAAAAAGAAAG	Sequencing
tnpA-Check-R	ACACGTTGAAAGCCAGAG	Sequencing
Ap-Check-F	TGGGCTATATTGAACTGGAT	Sequencing
PS1	AGGGCGGCGGATTTGTCC	Checking / sequencing (Silva-Rocha et al., 2013)
PS2	GCGGCAACCGAGCGTTC	Checking / sequencing (Silva-Rocha et al., 2013)
PS4	CCAGCCTCGCAGAGCAGG	Checking absence of pBAMDs plasmid backbone (Silva-Rocha et al., 2013)
PS5	CCCTGCTTCGGGGTCATT	Checking absence of pBAMDs plasmid backbone (Silva-Rocha et al., 2013)
PS6	GGACAAATCCGCCGCCCT	Checking absence of pBAMDs plasmid backbone (Silva-Rocha et al., 2013)

[a] Recognition sites for the restriction enzymes specified in the table are indicated in bold in the DNA sequence.

Table S2. Identification of the chromosomal locus of insertion of the *phaC1AB1* gene cluster from *Cupriavidus necator* in selected *Escherichia coli* JW2293-1 transconjugants[a].

Clone	Gene	Locus	Genome coordinate (bp)	Gene name and putative function	PHB accumulation level[b]
1	*ykgH*	*b0310*	324,097	Predicted inner membrane protein	+++
2	*betB*	*b0312*	326,772	Betaine aldehyde dehydrogenase	+++
3	*ligB*	*b3467*	3,817,737	NAD^+-dependent DNA ligase	+
4	*yagF*	*b0269*	284,181	CP4-6 prophage, D-xylonate dehydratase	++
5	*yadM*	*b0138*	152,722	Predicted fimbrial-like adhesin protein	+++
6	*ecpR*	*b0294*	310,037	MatA DNA-binding transcriptional dual regulator	++
7	*yadC*	*b0135*	150,509	Predicted fimbrial-like adhesin protein	+
8	*ydeT*	*b1505*	1,588,014	Predicted mercury detoxification protein	+
9	*pncA*	*b1769*	1,850,463	YdjE major facilitator superfamily transporter	++
10	*yahE*	*b0319*	335,614	Predicted protein of unknown function	+
11	*yadV*	*b0140*	155,969	Probable pilin chaperone similar to PapD	+

[a] The insertion sites were ascertained by means of arbitrary PCR with the oligonucleotides indicated in Table 2 in the main text, and the assigned function of the corresponding ORF is given according to the information available in the EcoCyc Database (Keseler et al., 2011).

[b] The poly(3-hydroxybutyrate) (PHB) accumulation level was qualitatively assessed by fluorimetry after viable staining with Nile red as indicated in the *Materials and methods* section in the main text. Individual clones were classified according to their PHB levels as + ≤ 15%, 15% < ++ ≤ 40%, and +++ > 40%; where the accumulation percentages correspond to the comparison with *E. coli* S17P (strain S17-1 λ*pir* carrying plasmid pAeT41, *phaC1AB1*$^+$; see Table 1 in the main text), used as a positive control in the polymer determination assay.

References

Brosius, J., Cate, R.L. and Perlmutter, A.P. (1982) Precise location of two promoters for the b-lactamase gene of pBR322. S1 mapping of ribonucleic acid isolated from *Escherichia coli* or synthesized in vitro. *J. Biol. Chem.* 257: 9205-9210.

Gibson, D.G., Young, L., Chuang, R.Y., Venter, J.C., Hutchison, C.A. and Smith, H.O. (2009) Enzymatic assembly of DNA molecules up to several hundred kilobases. *Nat. Methods*. 6: 343-345.

Keseler, I.M., Collado-Vides, J., Santos-Zavaleta, A., Peralta-Gil, M., Gama-Castro, S., Muñiz-Rascado, L., Bonavides-Martinez, C., Paley, S., Krummenacker, M., Altman, T., Kaipa, P., Spaulding, A., Pacheco, J., Latendresse, M., Fulcher, C., Sarker, M., Shearer, A.G., Mackie, A., Paulsen, I., Gunsalus, R.P. and Karp, P.D. (2011) EcoCyc: a comprehensive database of *Escherichia coli* biology. *Nucleic Acids Res.* 39: D583-D590.

Martínez-García, E., Calles, B., Arévalo-Rodríguez, M. and de Lorenzo, V. (2011) pBAM1: an all-synthetic genetic tool for analysis and construction of complex bacterial phenotypes. *BMC Microbiol.* 11: 38.

Silva-Rocha, R., Martínez-García, E., Calles, B., Chavarría, M., Arce-Rodríguez, A., de Las Heras, A., Páez-Espino, A.D., Durante-Rodríguez, G., Kim, J., Nikel, P.I., Platero, R. and de Lorenzo, V. (2013) The Standard European Vector Architecture (SEVA): a coherent platform for the analysis and deployment of complex prokaryotic phenotypes. *Nucleic Acids Res.* 41: D666-D675.
